# Dissecting Aging and Senescence—Current Concepts and Open Lessons

**DOI:** 10.3390/cells8111446

**Published:** 2019-11-15

**Authors:** Christian Schmeer, Alexandra Kretz, Diane Wengerodt, Milan Stojiljkovic, Otto W. Witte

**Affiliations:** 1 Hans-Berger Department of Neurology, Jena University Hospital, 07747 Jena, Thuringia, Germany; alexandra.kretz@med.uni-jena.de (A.K.); diane.wengerodt@med.uni-jena.de (D.W.); milan.stojiljkovic@med.uni-jena.de (M.S.); otto.witte@med.uni-jena.de (O.W.W.); 2 Jena Center for Healthy Ageing, Jena University Hospital, 07747 Jena, Thuringia, Germany

**Keywords:** aging, amitosenescence, brain, epigenetics, inflamm-aging, rejuvenation, replicative senescence, genetic program

## Abstract

In contrast to the programmed nature of development, it is still a matter of debate whether aging is an adaptive and regulated process, or merely a consequence arising from a stochastic accumulation of harmful events that culminate in a global state of reduced fitness, risk for disease acquisition, and death. Similarly unanswered are the questions of whether aging is reversible and can be turned into rejuvenation as well as how aging is distinguishable from and influenced by cellular senescence. With the discovery of beneficial aspects of cellular senescence and evidence of senescence being not limited to replicative cellular states, a redefinition of our comprehension of aging and senescence appears scientifically overdue. Here, we provide a factor-based comparison of current knowledge on aging and senescence, which we converge on four suggested concepts, thereby implementing the newly emerging cellular and molecular aspects of geroconversion and amitosenescence, and the signatures of a genetic state termed genosenium. We also address the possibility of an aging-associated secretory phenotype in analogy to the well-characterized senescence-associated secretory phenotype and delineate the impact of epigenetic regulation in aging and senescence. Future advances will elucidate the biological and molecular fingerprints intrinsic to either process.

## 1. Introduction

Advancing age has turned into the major risk factor for highly prevalent chronic and devastating diseases, including cancer, cardiovascular and neurodegenerative entities [1,2,3]. Classically, the aging process has been characterized by several candidate hallmarks comprising genomic damage and telomere curtailment, epigenetic alterations, dysregulation of proteostasis and nutrient sensing, mitochondrial dysfunction, stem-cell pool collapse, impaired inter-cell communication and cellular senescence (for a review see [4]). Thus, cellular senescence, first conceived almost 60 years ago and currently defined as a stress response by which cultured cells lose their proliferative capacity in an essentially irreversible manner, is regarded as a direct player in organismal aging in vertebrates [5]. Cellular senescence had been established mainly for replicative human diploid cells grown in vitro [6]. However, since the vast majority of cells in mammals are non-proliferating, the causative impact of replicative senescence in aging is still controversial, and current views are influenced by the relative success of senolytic approaches, which seem to halt and even reverse aging or initiate a state of rejuvenation [7,8]. A second important aspect in this context that emerged only recently as a result of the work of several labs, including our own work, suggests a proliferation-independent senescence-like mechanism in post-mitotic cells including neurons, which might also be involved in the organismal aging process [9,10,11]. Proof of such a phenomenon will conclusively entail the requirement for a revised definition of what cellular senescence subsumes and challenge a hitherto underestimated role of senescence mechanisms in neurodegenerative disorders and other pathobiologies originating from a post-mitotic environment. Thus, consolidation of post-mitotic cell senescence, a concept we term here ‘amitosenescence’ particularly in a neuronal context, would also strongly argue for the as yet controversially discussed functional interdependence of aging and senescence [12]. Proof of amitosenescence as a biological process would further amplify the contribution of cellular senescence to the aging process, in light of the fact that the majority of cells in the organism are nominally post-mitotic, and, in a final consequence, support the claim that aging is at least partially programmed.

**Figure 1:**
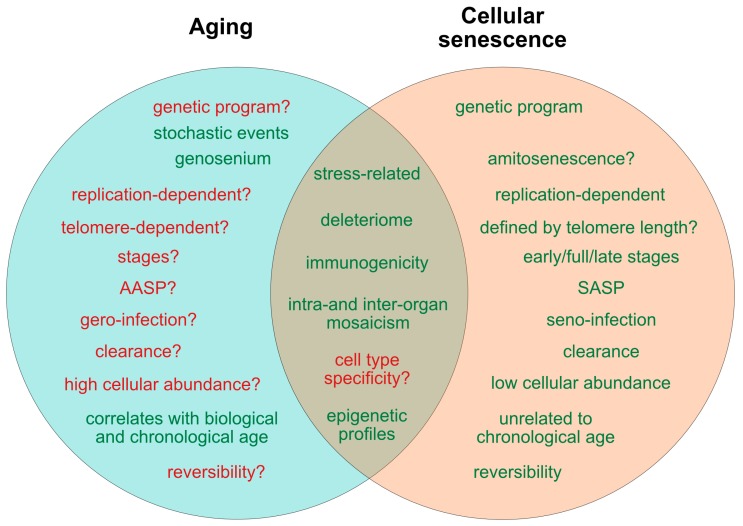
Current determinants and relevant open questions, marking the processes of aging and senescence as discussed in the text. Aspects represented in green are considered as broadly accepted or scientifically consolidated. Novel aspects that are yet unproven, or are under debate, are highlighted in red. SASP = senescence-associated secretory phenotype. AASP = putative aging-associated secretory phenotype as suggested in the text.

In spite of such conceptual dynamics regarding the molecular and cellular landmarks of senescence, and striking new evidence indicating that cellular senescence might play beneficial roles besides tumor suppression, its relevance in healthy and pathological aging as well as in life expectancy is still insufficiently understood. Improvements in this field inevitably will be linked to the establishment of well-accepted, comprehensive and clear definitions and functional stratifications concerning the biological differences between “cell aging” and “cell senescence”. However, both terms are often used indifferently and with low stringency and, thus, can provoke crucial misinterpretations regarding their individual and intercalated roles. Therefore, in the following sections, we will discuss recent and novel concepts on aging and senescence, and report evidence revealing that, although cellular senescence contributes to organismal aging, it appears to be independently regulated in many aspects. In the light of aging being understood as a primarily organismal phenomenon, we will, however, discuss both processes at the cellular level whenever applicable and compare their roles on the basis of factor-specific opposition in terms of conceptual aspects, molecular regulation and biological consequences (for an overview see Figures 1 and 3). By providing such an overview, we aim to achieve a clearer outline of our current scientific definition and understanding of aging and senescence.

## 2. Current Concepts of Aging and Senescence

The processes driving the progressive decline of functional performance during aging are yet not fully understood. Accordingly, the term ‘aging’ is interpreted ambivalently in the field, and effectors of aging remain a hotly debated issue. Moreover, there is still controversy regarding the interrelation between aging and disease and whether aging reflects a disease state or not [13]. Generally accepted is the basic view that aging subsumes an organismal phenomenon involving an increased chance of dying and/or decreased functionality over time [14]. Although the same definition is applicable at the organ and tissue levels, defining aging at the cellular and molecular levels has been challenging [14].

### 2.1. Is Aging Programmed?

In the last 50 years, several theories have been proposed to explain the nature and control of aging. Some of them include: 1) the evolutionary theory based on the concepts of deleterious mutational accumulations developed by Peter Medawar in 1952 [15] and antagonistic pleiotropy by George Williams in 1957 [16], which states that specific alleles and mutations that display either positive or no effects on fitness in early development can be detrimental in later life as natural selection fails to remove them, thus causing aging; 2) the free radical theory proposed by Denhan Harman in 1956 [17], suggesting that detriment to cellular components by successive accumulation of oxygen species overwhelms the anti-oxidative mechanisms; 3) the programmed theory proposed by Valter Longo and colleagues in 2005 [18], based on insights by August Weismann in the 19th century, which explains aging and death as a result of a genetic program that evolved to benefit future generations; and 4) the hyperfunction theory stated by Mikhail Blagosklonny in 2008 [19], which suggests that sustained hyperactivity of genes during the reproductive age window causes a state of cellular hypertrophy that results in aging, but is not primarily related to molecular damage.

**Figure 2:**
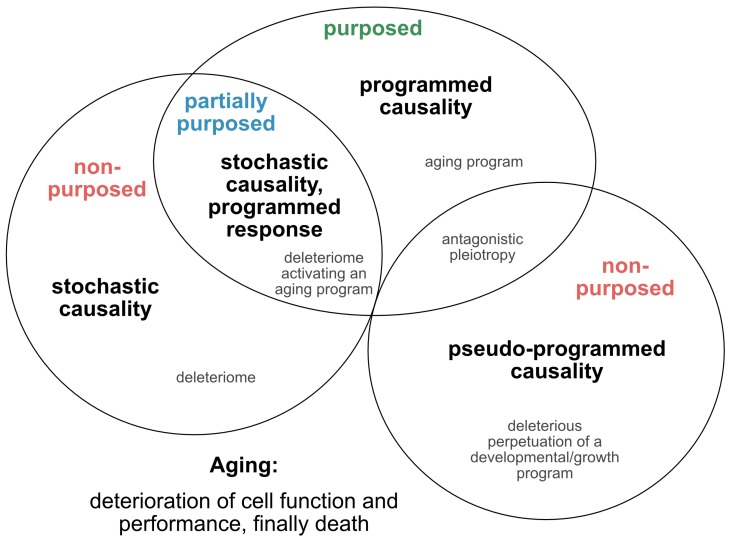
Theories on the causality and purpose of aging. Graphically summarized are four contrasting concepts crystallized from current evidence addressing the inductive driving force of aging. Apart from a stochastic deleteriome, there are arguments for a pseudo-programmed, programmed or at least partially programmed nature of aging.

Such approaches, which provide tentative explanations for the causes and mechanisms of the aging process, have continued to evolve and are being translated into novel concepts, fueling the yet ongoing debate over whether aging is programmed, or merely a purposeless, unintended and stochastic accumulation of deleterious events that reduce the performance of an organism (Figure 1). In an attempt to reconcile such theories and pave the way for a directive for promising future concepts, we here extract and re-define the following four essential points of view (Figure 2): **1) the stochastic causative approach** propagates the concept that aging arises from the stochastic time-dependent conglomeration of genotoxic events, oxidative stress, loss of repair capacities and other hallmarks that account for cellular deterioration with chronological aging [4]. Whether the development of such a deleteriome is putatively causal to the aging process [20], as already speculated by Harman in 1957, and whether it is devoid of a biological purpose, or follows the evolutionary aim to prioritize young over old organisms to support biological fitness and speed positive selection, are still a matter of debate; **2) the pseudo-programmed causative approach** represents the position coined by Mikhail Blagosklonny in 2013, that aging ‘is not and cannot be programmed’ [21], as it reflects the continuation of an embryonic program into adulthood, which cannot be powered off but loses purpose with time. Such a pseudo-program thus shares the same cascades integral to growth and development and drives geroconversion, characterized by growth without cell division, and cellular senescence, the second being regarded as a continuation of differentiation [21]. In conclusion, aging is, therefore, assumed to originate from a genetic pseudo-program that is reminiscent and a relic of a developmental growth program, which, however, is independent from a deleteriome. If such a concept holds true, aging might neither be programmed, nor represent a pure stochastic and random accumulation of events that provoke aging and age-related pathologies, and finally culminate in death. In essence, it mirrors antagonistic pleiotropy, but without the claim of being purposed; **3) the programmed causative approach** suggests that aging is partially, or even fundamentally programmed [22]. Likewise, it was recently illustrated that aging might be ruled by a global, unique, but pleiotropic genetic motif. This highly conserved, transactivating binding motif is strongly associated with transcriptional changes culminating in the process of age-related genetic reprogramming [23,24]. In analogy, reversion of this program, which is essentially nuclear factor kappaB (NF-κB)-mediated, has been illustrated to counteract molecular- and cellular-aging phenotypes [23,24], supporting the notion that the aging process is not self-sustained once initiated by certain genetic mechanisms, but requires a pro-active continuation of a genetic control mechanism. Moreover, the discovery of so-called ‘longevity genes’ and of strategies interfering with, e.g., sirtuins, insulin/insulin-like growth factor-1 (IGF-1) and calorie intake, all of which potently counteract the physiological aging process in different paradigms [25], argues in favor of aging being, at least to some extent, a genetically guided process [23,26,27]. In support of this, longevity factors including Sirtuin 1 (Sirt1) and the Forkhead box protein O (FoxO) are reported to antagonize transcriptional profiles related to the transactivating binding motif mentioned above [26,28,29]. The genetic motif described is also essentially involved in the processes of immuno-aging and age-related inflamm-aging [26,27] and constitutes a main molecular driver of the senescence-associated secretory phenotype (SASP) [30]. Moreover, as it also governs organismal development as a whole [31,32], this concept supports the aforementioned notion of aging representing a ‘quasi-program’ intricately linked to the genetic program underlying growth and development [21]; This motif in terms of NF-κB has recently been highlighted as a downstream target of the cyclic guanosine monophosphate–adenosine monophosphate (GMP–AMP) synthase (cGAS) that controls, in alliance with the class of ‘Stimulator of interferon genes’ (STING), an innate pro-inflammatory immune response elicited by the highly immunogenic nature of ectopic DNA species. In analogy to cancer cells, senescence cells activate the cGAS-STING pathway in response to the occurrence of cytoplasmic heterochromatin, which, in turn, activates paracrine SASP [33,34] and NF-κB that drives itself the SASP and sustains inflamm-aging. Therefore, considering that an innate cGAS-STING immunogenicity path is engaged in the control of senescence might represent a novel direction to counteract senescence. How far hyperactivation of this pathway might bear the risk for tissue damage and chronically sustained inflammation still has to be defined; **4) the stochastic causative, but programmed response approach;** based on current knowledge, it is well-accepted that stochastic alterations at the cellular level, e.g., DNA damage, free radical accumulation and mutations, induce compensatory mechanisms to reduce the impact of multiplied damage on an individual’s fitness, health and lifespan. Therefore, lifespan is limited by the capacity to counteract condensed damage and dysfunction. Accordingly, aging will originate from that part of the deleteriome that cannot be neutralized by the compensatory mechanisms individually activated—a process that is initiated with the commencement of life. Counterbalancing mechanisms will work differently in each individual due to variable genetics, epigenetic background, environmental and other stressors and susceptibility factors. One novel aspect among these vulnerability criteria comprises the recent illustration that most organs are composed of an assortment of cells, protein complexes and individual proteins of strikingly different ages, regardless of whether they are quick or slow to regenerate [35,36,37]. Such age mosaicism in the proteome of cells and their structural components assumes exceptional importance in post-mitotic cells as their nucleus will not be replaced over lifetime and thus directly links nucleosome protein turnover to nuclear integrity, chromatin organization and gene transcription. Thus, aging is the failed purpose to counteract damage, and this failure causes functional decline, pathology and death. Factors causative in the initiation of aging are stochastic, but aging-associated response mechanisms such as DNA damage response (DDR), reactive oxygen species (ROS) neutralization, metabolic adaptation, senescence or apoptosis are programmed. This approach also suggests that, although propagation of aging at the organismal level might be irreversible, the kinetics can be decelerated, achieving a partial and ‘relative rejuvenation’ at the cellular, tissue and even organ level.

In our view, recent evidence that senescence is based on an unterminated developmental growth program [24] and the finding that the concept of post-mitotic senescence requires the activation of expansion, or ‘growth’ factors as a second hit [38], favor the assumption that aging underlies a grating of genetic determination similarly to what is summarized above under the pseudo-programmed causative approach (Figure 2).

### 2.2. Single Nucleotide Variants as Genetic Markers Supporting Programmed Aging?

A recent study by Lodato and colleagues, based on a single-cell whole-genome sequencing approach, indicated that in humans terminally differentiated post-mitotic neurons agglomerate somatic mutations with age, thereby displaying age-, region- and disease-specific molecular signatures [39]. Further characterizations of these single nucleotide variants (SNVs) that accumulate almost linearly with aging, a state termed genosenium, and of the even higher abundance of SNVs under neurodegeneration might have the potential to identify genetic markers accessible for therapeutic intervention. Thus, the finding that SNVs and several genes can influence the progression of aging and age-related disorders and impact life expectancy, will surely encourage further unbiased whole genome approaches empowered by enlarged subject numbers. Such studies will putatively relieve the search for a static and universal aging program by the identification of dynamic genetic risk and individualized susceptibility profiles. Such efforts will equally help to better define which concept reflects the nature and biology of aging, and reveal whether there might operate yet undiscovered molecular players, beyond the prolongation of developmental cascades or patterns of SNVs, that drive a pseudo-program of aging. An interesting new step in this direction will be to clarify if such SNVs also support the notion of aging involving genetic fingerprints of growth and development. Accordingly, tracing spontaneous somatic mutations that occur early in life and spread via clonal expansion in replication-competent neural cell entities, as it has been realized by Lee-Six and colleagues in a hematopietic cell context [40] might further improve our understanding of the genetic influence on aging.

### 2.3. Senescence—A Programmed Process

By contrast, it is accepted that cellular senescence is executed in the framework of sequential and tightly orchestrated stress-induced effector programs involving the down-regulation of Cdks, up-regulation of CdkIs, increased metabolic activity, activated DDR pathways and ultimately the induction of apoptotic effectors [41,42]. Moreover, comparative transcriptome analyses have allowed for the identification of senescence-specific genes including *PVRL4, PRODH, LY6D, DAO, EPN3,* and *GPR172B*, all of which are dependent on p53 [43]. In particular, *PRODH, DAO*, and *EPN3* were shown to promote senescence. In contrast, ectopic expression of GPR172B inhibited senescence. The obvious particular role of p53 is supported by a recent computational-assisted work by Kirschner et al. that identified a comprehensive and self-regulatory network of p53-dependent downstream targets, which are specific for acute and chronic kinetics of a cell type-specific p53 regulome [44]. Also, removal of senescent cells under physiological conditions is obviously a programmed process involving both cellular and humoral constituents of the immune system [45].

Despite such a general consensus about senescence underlying a timely and molecularly determined program, the biological importance of the genetic factors engaged is still vague and ill-defined with regard to cellular, tissue and organ specificity. This is in striking contrast to the detailed characterization of other processes such as development and apoptosis that are much better elucidated in terms of kinetics and effector cascades.

### 2.4. Role of Cell Replication, Telomere Shortening and DNA Integrity for Aging and Senescence

**Figure 3:**
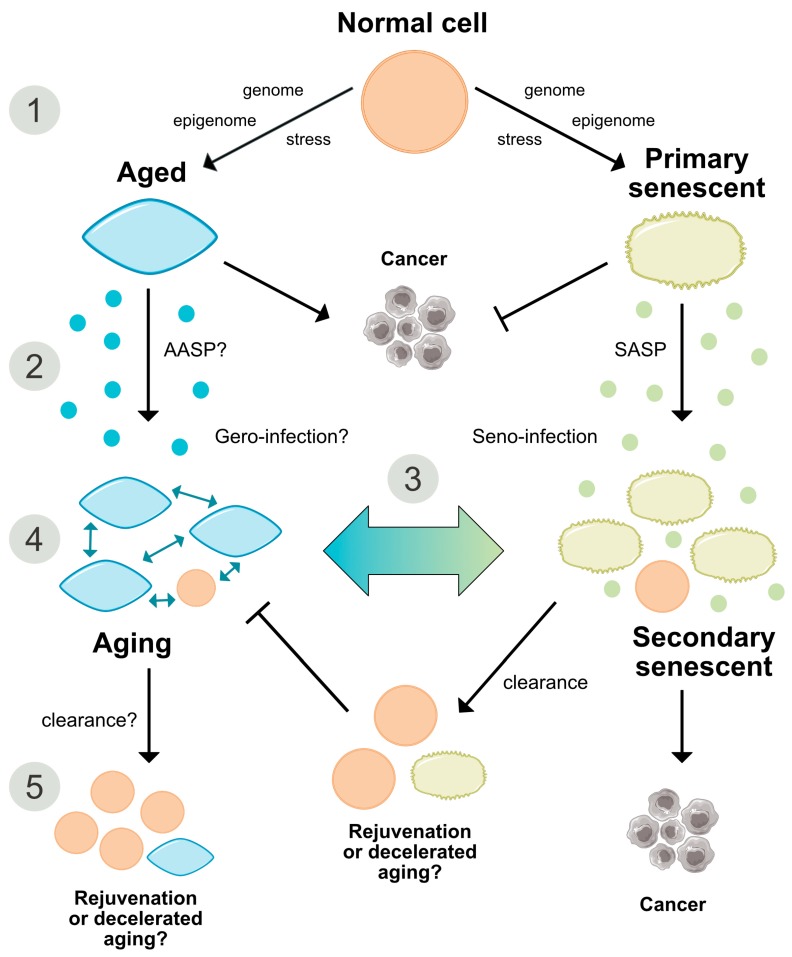
Comparative representation of the aging and senescence processes highlighting different levels of interaction and putative sites of interventions. (**1**) As discussed in the text, causative mechanisms of aging are still not well understood, however, multiple factors including genetic, epigenetic and stress-related effects seem to have an orchestrated role in the progression of aging. Senescence on the other hand, is seen as a programmed response to different kinds of stressors, which proceed in defined stages. Whether, in analogy, aging also follows a defined program or sequential stages is not known. (**2**) Senescence involves autocrine and paracrine factors, which are responsible for a ‘seno-infection’ or bystander effect in neighboring cells. There is currently no direct evidence for a similar factor composition propagating the aging process via a kind of ‘gero-infection’. (**3**) Accumulation of senescent cells has been described as a hallmark of aging; however, whether they are a causative factor or a consequence of tissue and organismal aging is still unknown. As discussed in the text, it appears possible that aging and senescence mutually influence each other through positive feedback at this level, leading to accelerated tissue damage and aging. (**4**,**5**) Clearance of senescent or aging cells might constitute putative targets for interventional approaches aimed to reduce or reverse the impact of aging and improve cell and tissue homeostasis by inducing a ‘rejuvenation’ process. SASP = senescence-associated secretory phenotype. AASP = putative aging-associated secretory phenotype as suggested in the text.

Apart from the controversy on the presence or absence of a putative underlying genetic program connecting aging and senescence, there are several other determinants that draw a picture in favor of aging and senescence being interacting or intercalating, but independent processes (Figure 1 and Figure 3). Senescence was initially described for replicative cells and is still mainly linked to replication-competent cell moieties, whereas there is currently no clear association between the aging process and the turnover rate of cells in different tissues and organs [46,47], a fact that seemingly underlines the independence of the two processes. On the other hand, primarily post-mitotic organs such as the adult brain are equally subjected to age-related dysfunction as replicative organs [48]. Thereby, the presence of a proteome that contains a range of extremely long-lived proteins susceptible to acquiring time-dependent malfunctions might contribute, together with other factors, to explain age-related functional decline in the central nervous system (CNS) and other post-replicative tissues [36,49,50]. Hence, the novel finding of senescence engaging post-mitotic moieties [10], the discovery of patterned SNVs in aged neurons [39], and the identification of inter- and intracellular age differences of individual proteins [35,36,37] suggests that senescence or a senescence-like deregulation is currently underestimated in its impact on aging, and might occur in post-mitotic tissues even at a subcellular scale.

Similar considerations apply to the question of how far replication-independent telomeric alterations are relevant for both senescence and aging, and how far replication-associated telomere curtailment propagates either process (Figure 1).

Increased telomere shortening with each cell division cycle is assumed to belong to the key drivers of cellular senescence as soon as a critical length is attained, thereby initiating a DDR entailed by ultimate proliferation arrest [51]. By contrast, naïve telomere length in different species and even inter-individually can greatly vary independently of age, and is generally weakly correlated with their lifespan [52,53]. Therefore, the role of absolute telomere length in aging is still a matter of debate. Alternatively, we recently gathered evidence that the rate of shortening rather than the absolute telomere length might be a better predictor to connect telomeres to the aging process [9]. In analogy, studies performed in proliferation-competent cells emphasize that the main effector responsible for the activation of the p53 and p16^Ink4a^-Rb pathways involved in telomere-directed replicative senescence is not the loss of telomeric DNA but rather the impaired function of individual telomeres that become extremely short [54,55].

In expansion to the aforesaid in the context of amitosenescence, there is novel evidence that stress-related cellular senescence might be telomere-related also in post-mitotic cells, and that this process plays critical roles during normal aging, and under pathological conditions [10,11,56]. Such a phenomenon appeared even independently from telomere shortening since it could not be reversed by exogenous expression of telomerase [57,58,59]. By contrast, obviously it originates from a DDR activated at deeper sites of damaged telomeres [57,58,59].

Given that the DNA damage load in post-mitotic, senescent-like cells arises from a second step-incompetence to perform homologous recombination as an error-free repair mechanism due to their ultimate cell cycle arrest, the question arises of how neurons sustain their vitality and functional state throughout lifetime. This obvious genomic threat is pronounced by their high DNA-damaging ROS production and transcription rate, features that further contribute to DNA disintegration. In accordance, post-mitotic neurons display a broad range of senescence-related markers including DNA strand breaks, increased p21^Cip1^ expression representing an essential signal transducer linking DNA damage response to a senescence-like phenotype, elevated base pair oxidation, heterochromatinization, activated p38 mitogen-activated protein kinase (MAPK) and augmented IL-6 levels, as well as senescence-associated-β-galactosidase (SA-β-Gal) expression [10]. Notably, the frequencies of such senescence-like hallmarks in neurons strongly increase with age. The complex susceptibility of neurons towards genomic DNA instability might be aggravated by the aforementioned fact that telomeres in general constitute a persistent site of DDR at deeper aspects of their repetitive sequence, irrespective of critical end cap erosion and replication rate [57,58]. In order to elucidate the telomere length dynamics in post-mitotic neurons, we evaluated changes in relative telomere length in the aged mouse brain using flow-cytometry-based in-situ hybridization (Flow-FISH) and real-time quantitative polymerase chain reaction (RT-qPCR)-based methods and found that both cell cycle-dependent and -independent telomere shortening accompanies the murine brain aging process, with post-mitotic neurons being direct contributors to brain-intrinsic telomere curtailment [9]. The mechanisms responsible for telomere shortening in non-proliferative neurons are still not clear as, e.g., brain telomerase activity remained unaltered with aging [9]. Whether neuronal telomere attrition in the aged CNS corresponds to a senescent phenotype at the cellular level still has to be elucidated.

A recent study by Moreno-Blas et al. describes dysfunctional autophagy as an alternative effector involved in senescence induction in post-mitotic neurons [60]. In the study, long-term cultured neurons acquired several senescence-associated features including expression of SA-β-Gal, p21^Cip1^ and γH2AX due to impaired autophagy, and developed a SASP-like phenotype able to induce paracrine premature senescence. Since it is known that autophagy loses efficiency during aging [61], such findings raise the question whether altered autophagy might constitute a key element in the crosstalk between aging and senescence. Re-establishment of autophagy might therefore represent a putative therapeutic target for senescence- and aging-associated pathologies. Results from this study contradict the later mentioned study by Bussian et al., who could not identify a process of neuronal senescence [62]. The discrepancy might arise from alternative timing used for the geroconversion of the different cell types present in the brain [60].

The question arises of whether such a senescence-like phenotype in post-mitotic cells, irrespective of the underlying inductive mechanism, will serve as a process to prevent pathology. As a case in point, ganglion neurons directly opposed to blood vessels that degenerate in retinopathies do not die as expected after deprivation from nutrients and blood supply but, instead, adopt a senescent-like state that appears to protect them from apoptosis [63]. Importantly, under these conditions, senescent-like retinal neurons secrete the neurovascular guidance factor SEMA3A that is known to trigger paracrine senescence in vitro in neurons, macrophage-like cells, and endothelial cells [63]. The ultimate fate of cells with features of amitosenescence remains elusive, and it is still unknown whether they are eventually cleared or will also accumulate with aging.

Such considerations are also relevant with respect to the aforementioned ‘deleteriome’ concept [20], which refers to the timely accumulation of different entities of cellular insults, with the strongest contribution mediated by DNA damage, including telomere site-specific demise. Although each cell in the organism harbors DNA alterations, only a limited fraction will manifest as senescent, and thus the relative contribution of senescence to the process of aging is still not well understood. However, as brought into focus recently by Ogrodnik and colleagues [64], senescent cells appear to possess an extraordinarily high amount of DNA injury at the single-cell level, as featured by the burden of irreparable intra-telomeric DNA damage sites [58,65]. Thus, although few in absolute number, the relative impact expressed by DNA damage events might be potentiated in the fraction of senescent cells and thereby drive aging. This hypothesis raises the question of how long such high and irreparable levels of DNA injury might be tolerated prior to the induction of apoptosis, or in how far these senescent cells accumulate with aging, as they are, at least to a certain degree, protected from apoptosis, and thereby might drive detrimental effects. Moreover, although the amount of DNA injury might correlate with the manifestation of a senescent phenotype, the inference that the severity grade of DNA injury in a single cell imposes a stronger impact on the aging process still requires confirmation.

### 2.5. Role of the Immune System in Cellular Senescence and Aging

An intricate interrelationship between inflamm-aging and immuno-senescence during aging has been proposed, with both immune processes influencing each other [66,67]. Whereas inflamm-aging indicates the increase in pro-inflammatory activity of the innate immune system represented by activated macrophages or microglia, immune-senescence describes the lower precision and activity of the adaptive immune system at higher ages, primarily caused by thymus involution and alterations in T-lymphocyte subsets [68]. In spite of a mutual influence and regulatory similarities, the two processes address different immunological aspects [69]. Attributing individual aging and senescence parameters to either branch will further refine our understanding in the field.

Likewise, senescent cells displaying the SASP, which stimulates both innate and adaptive immune mechanisms, might be particularly susceptible to concentrate in the brain, as the immune reactions relevant for their elimination are excluded from the immune-privileged CNS [70]. Whether there is an innate immune-mediated mechanism involved in the clearance of senescent-like cells in the CNS remains to be addressed. In addition, senescent cells seem to be able to escape immune elimination, e.g., via human leukocyte antigen (HLA)-E-mediated natural killer (NK) and CD8^+^ cell inhibition [7,71] that is stimulated by paracrine release of pro-inflammatory cytokines. While accumulation of senescent cells has been interpreted to trigger aging, it could also mean that senescent cells result from the aging process [72].

### 2.6. Are Both Senescence- and Aging-Associated Alterations Mediated by a Cell-Specific Secretome?

Senescent cells secrete many factors well characterized to coin the SASP [12,73] (Figure 3). SASP factors comprise several interleukins including IL-6 and IL-1, and chemokines such as CXCL-4, -5, -6, -12 and CCL-2, -3, -7, -8, -13, -16, -20 and -26 [60,73]. The SASP is also mediated by other pro-inflammatory cytokines such as the colony-stimulating factors (CSFs), extracellular proteases (MMPs) such as MMP-1, -3, and -10, serine proteases [74], fibronectin [75] and nitric oxide and reactive species [76,77]. The signature combination of factors released by senescent cells depends on the cell type and the inducer of senescence [60,78]; however, many key effectors are shared. These include the aforementioned NF-κB and CCAAT/enhancer-binding protein ß [79,80,81].

The SASP acts as a tumor suppressor [82], however, it is also connected to tumor induction by generating a pro-inflammatory tumor-favoring microenvironment [83]. Moreover, as indicated in oncogene-induced senescence (OIS), the SASP propagates paracrine secondary senescence in neighboring cells (Figure 3). Thereby, Teo and colleagues showed that primary and secondary senescent cells represent two different transcriptional endpoints, the first characterized by Ras stimulation and the second by Notch activation [84]. The reduction of such a senescent spread might be relevant for the development of therapeutic approaches. However, direct proof of this tissue-compromising bystander effect and its attribution to a responsible cell type under in vivo conditions is still required (Figure 3).

**Figure 4:**
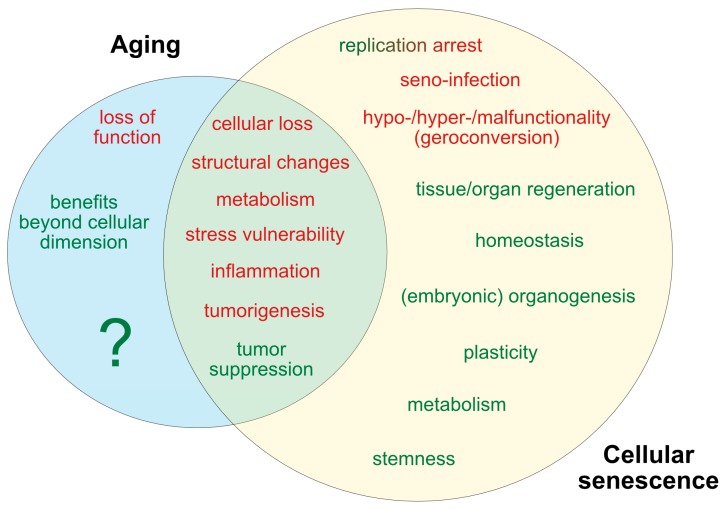
Pathological and beneficial functions of aging and senescence, according to current knowledge. In red are represented pathological consequences and in green beneficial functions of aging and senescence. The impact of aging has mainly been described at the organismal level, since a complete cellular functional profile has not yet been established. Accordingly, whether beneficial consequences of the aging process exist at the cellular level is unclear.

Induction of the SASP involves activation of several signaling pathways such as DDR, p38 MAPK, and mechanistic target of rapamycin (mTOR) [73,79]. In particular, growth-promoting, mitogen-/nutrient-sensing pathways such as mTOR drive the process of geroconversion, which follows or precedes cell cycle arrest [85,86] and further supports our favored hypothesis that aging requires a sustained growth program mirroring development (Figure 4). Thereby, gerogenic conversion or ‘geroconversion’ is defined as a form of growth during cell-cycle arrest, which translates reversible into irreversible arrest, a hallmark of senescence. It implicates a hyper-trophic, hyper-secretory, pro-inflammatory and hyper-/malfunctional cellular or organ state that predisposes to age-related disorders. Importantly, structural hypertrophy and hyperfunctionality have also been considered the most striking features of cellular aging [19]. Interestingly, although p16^Ink4a^ efficiently induces senescence-associated cell-cycle arrest, it seems not to be critical for SASP activation, since increased exogenous expression of this tumor suppressor leads to lower induction of SASP factors relative to other senescence inducers [73,87]. This would suggest that the SASP is not necessarily a consequence of p16^Ink4a^ activation or senescence per se, but rather a cellular response to damage, which is independent of replicative arrest. Underlining this notion, endogenous cytoplasmic chromatin fragments derived from damaged, senescent nuclei upon LaminB1-dependent disruption of the nuclear lamina are sensed by the pattern recognition receptor cGAS, whose activation in turn mediates the production of SASP components in a STING-dependent fashion [33,34]. Triggered by different senescence stimuli inducing DNA damage, oxidative stress or OIS, the p16^Ink4a^-independent cGAS-STING pathway proved to be necessary for the induction and perpetuation of the SASP and, therefore, might contribute to the immune surveillance and probably to elimination of damaged and dysfunctional cells [33,34].

A study by Hudgins and colleagues found dynamic changes in the expression of established senescence markers, including p16^Ink4a^, p21^Cip1^, and SASP factors in different tissues including the brain hypothalamus, with age [88]. Importantly, the hypothalamus, suggested to be a superordinate pacemaker in the aging process, mediates its functional role through NF-κB signaling, an aforementioned effector in cellular senescence and putatively programmed aging [23,24,89]. In the hypothalamus, IL-1β, Mmp12, and Cxcl1 expression was increased in 30-month-old relative to 4-month-old mice. Surprisingly, IL-6 expression was decreased, p16^Ink4a^ expression was not detectable and p21^Cip1^ expression was unchanged [88]. The absence of prototypical markers in spite of striking evidence for senescence-like cellular phenotypes in CNS tissue [10,11,56] further emphasizes the need for adequate senescence markers specific to the conditions of amitosenescence.

To date, it is still not known whether the aging process is also influenced by a SASP-like autocrine and paracrine profile that might determine the recruitment and instruction of immune cells and the aging process itself. Importantly, experiments using heterochronic parabiosis showed that exposing young mice to an old systemic environment or plasma from old mice impairs brain function and neurogenesis. Furthermore, this effect was associated with increased levels of various factors, including chemokine CCL11/Eotaxin and β_2_-microglobulin [90,91]. On the other hand, exposure of aged mice to young blood late in life improved brain function and had a ‘rejuvenating effect’, supporting a role of paracrine factors in the aging process [92]. There is also growing evidence for a role of the growth hormone (GH)-IGF-1 axis in aging and age-related disease manifestation [93]. The peptide hormone somatotropin (STH), or GH, pulse-released from the anterior pituitary gland is under negative regulatory feedback of IGF-1, somatostatin and insulin. The IGF-1 signaling pathway controls multiple cellular functions relevant in aging, such as cell-cycle progression, apoptosis, immune cell propagation and chemotaxis [94], entailed by the activation of several intracellular cascades including PI3K, MAPK, mTOR and FoxO, the latter two of which are well-established in their influence on the aging process [95,96]. Additionally, IGF-1 is related to subclinical inflammation, as recently demonstrated in human peripheral blood mononuclear cells [97]. Human individuals with genetically determined GH receptor insufficiency (Laron syndrome) exhibit a significantly lower risk of developing cancer [98] and show a prolonged lifespan at least in females [99]. Moreover, genetic mutations conferring partial IGF-1 resistance are enriched in individuals with extended life expectancy, and low IGF-1 levels correlate with reduced death risk and improved performance in the daily life activity of nonagenarians [100,101,102] (for a review, see [103]). Thus, although not systematically proven, the chronological changes and age-related decline in the highly conserved GH/IGF-1 pathway over lifetime might contribute to a SASP-like counterpart that influences aging at the secretory level. The characterization of such a putative aging secretome or ‘aging associated secretory phenotype’ (AASP), would further aid in segregating senescence from aging, and could also represent a putative target to reduce the progression of aging-associated alterations (Figure 1 and Figure 3).

### 2.7. States of Aging and Senescence

Cellular senescence has been shown to proceed through three phases referred to as early, full and late stages [104]. The early stage is characterized by morphological and functional alterations in response to stressors, the second phase is dominated by the SASP response, and a late stage displays the upregulation of L1 (LINE-1) retrotransposable elements and an IFN-I response [42,105]. Activation of retrotransposons has been proposed as an important element of sterile inflammation, a hallmark of aging, suggesting retrotransposition as a factor that might further link aging and senescence. The copy number of L1 transposons is particularly high in the CNS as compared to other somatic tissues [106]. Therefore, the apparent contribution of the retrotransposon-derived transcriptome to CNS dysfunction and neurological disease manifestations [107,108] mediated, e.g., via their intrinsic gene regulatory functions, or their de-repression due to impaired RNA and epigenetic control mechanisms and disturbed DNA repair under pathological conditions (for review, see [109]), might also arise from the propagation of senescence-like processes. In support of this, transposable elements, which are broadly targeted and silenced by the multifunctional RNA binding protein TDP-43 are well-known to be de-repressed in TDP-43 pathology-associated neurodegeneration, such as in the spectrum of age-related fronto-temporal lobar dementia (FTLD) and amyotrophic lateral sclerosis (ALS) [108]. Similarly, in *Drosophila* transcript levels of *R1/R2* and *gypsy*, representing LINE-like and long terminal repeat (LTR) elements, respectively, are increased with healthy brain aging, and genetic manipulations unleashing transposon expression impair CNS memory functions and shorten lifespan in the fly [110]. Beyond the aforementioned SNVs and epigenetic regulation, intra-individual variability in the spatial distribution and rate of retrotransposons and their activation might therefore also provide a genetic source to explain the cellular mosaicism attributable to aging and senescence. Moreover, since adult neurons are shown to actively propagate retrotransposition [111], it is tempting to speculate that these mobile elements might also contribute to the phenomenon of amitosenescence, e.g., through DDR activation. Thus, apart from quantitative considerations, resolving both the structure and genomic location of retrotransposon insertion and transcription might gain relevance to understand their function in senescence, aging and age-related pathology.

As delineated in the context of the SASP, senescent cells can be separated between primary and secondary, i.e., ‘infected’ senescent cells. Whether such differentiation, apart from representing a local propagation mechanism, also implicates other biological consequences, e.g., with regard to cellular survival, tumor growth and molecular signaling, still has to be explored [84]. In an analogy, it would be interesting to determine whether similar early, intermediate and late stages exist for aged cells in vivo, and whether such stage classifications would be suitable to separate reversible from irreversible aging processes (Figure 1).

## 3. Markers of Aging and Senescence

To identify, follow and quantify senescence, specific biomarkers have to be established. Similarly, although addressed with less frequency in scientific approaches, markers for aging are also defined.

Several morphological and molecular criteria have been used to identify proliferation arrested senescent cells undergoing either replicative senescence (RS) or stress-induced premature senescence (SIPS): 1) altered (often enlarged, flat, multivacuoled and multinucleated) cellular morphology, 2) increased activity of senescence associated-β-galactosidase (SA-β-Gal), 3) accumulation of DNA damage foci; 4) accumulation of senescence-associated heterochromatic foci (SAHF), linked to histone variants such as macroH2A (mH2A) [10] and other chromatin modifications, 5) accumulation of promyelocytic leukemia protein (PML) nuclear bodies and so-called ’DNA segments with chromatin alterations reinforcing senescence’ (DNA-SCARS), 6) induction of the SASP, 7) increased levels of Cdk inhibitors, p16^Ink4a^/Rb and/or p53/p21^Cip1^ [112,113].

Interestingly, p16^Ink4a^ is also considered one of the best aging markers as it is suppressed in early embryogenesis and progressively induced during aging [114], further providing a link for a putative functional interdependence between aging and senescence. In the context of aging, reduced expression of p16^Ink4a^ has been associated with the reversal of age-associated functional decline. In p16^Ink4a^-deficient mice, the decline in neural progenitor cell proliferation and function in the subventricular zone and olfactory bulb normally observed with maturation and aging was reduced without affecting progenitor function in the dentate gyrus or enteric nervous system, suggesting a causative role for p16^Ink4a^ in the age-associated decline in neurogenesis [115]. Moreover, increased p16^Ink4a^ expression prevented the activation of aged quiescent stem cells in the dentate gyrus by physical activity and this was partially reversed by motor training in p16^Ink4a^ null mice [116].

At the intersection of aging and senescence, long-term clearance of p16^Ink4a^-positive senescent cells prevented some age-related pathologies and increased the lifespan in a mouse model of accelerated aging [117,118]. In addition, a study by Bussian and colleagues indicated that elimination of p16^Ink4a^-positive astrocytes and microglia in the *MAPT*^P301S^*PS19* mouse model of tau-associated cognitive impairment prevents hyperphosphorylation of soluble and insoluble tau forms, gliosis and also cognitive decline [62]. Moreover, Chang and colleagues developed a drug, the BH3 mimetic ABT-263 that blocks the interaction between antiapoptotic Bcl-2 proteins and their targets. ABT-263 selectively eliminates senescent cells in healthy aged mice by apoptosis, thereby improving the regenerative capacity of aged hematopoietic cells and muscle stem cells [119]. Such findings clearly suggest a causative role of cellular senescence in aging. A study by Hall and colleagues reported that macrophages express p16^Ink4a^ and SA-β-Gal as part of a reversible response to physiological immune stimuli rather than through senescence, indicating a senescence-independent increase of p16^Ink4a^-expression [120]. Moreover, proliferating mesenchymal cells of young mice lacking other properties of cellular senescence were found to be highly positive for p16^Ink4a^ [121]. In line with these findings, we observed significant expression of p16^Ink4a^ both in microglia from newborn and young adult murine brains [122]. Therefore, it is generally relevant to differentiate between p16^Ink4a^ senescence-independent functions and its indication of programmed developmental senescence. Furthermore, artificial overexpression of the *Ink4/Arf* locus resulted in reduced expression of aging markers and extended lifespan, suggesting a rather anti-aging effect of p16^Ink4a^ [114,123,124]. It is important to underline that discrepancies between the studies might arise from approach-related peculiarities. Overexpression of p16^Ink4a^ was achieved by insertion of two additional copies of the *Ink4/Arf* locus that codes not only for p16^Ink4a^, but also for p19^Arf^ [123]. It is conceivable that the outcome from some of the studies is dependent on whether physiological or non-physiological conditions were used.

DNA damage strongly propagates senescence and is causal in accelerated aging. Likewise, several hereditary syndromes implicating DNA repair deficiencies are linked to premature aging [125]. Thereby, both increased frequency of DNA damaging insults and impaired repair capacity have been shown to accompany the expression of SA-β-Gal in primary cultured neurons [126], although markers for DNA strand breaks such as γH2AX and 53BP1 do not always co-localize with SA-β-Gal [127]. Therefore, using SA-β-Gal as an indicator of cellular senescence might be critical under certain conditions, in particular in a neuronal setup [127], where it also reflects lysosomal activity or other states unrelated to cellular senescence [128].

In conclusion, although frequently used, both p16^Ink4a^ and SA-β-Gal are not exclusive markers for senescence and aging but can indicate other biological functions and even anti-aging properties, dependent on the cellular context. Such data also suggest that senescent cells may not be the only populations associated with age-related frailty. This reflects the urgent need for refinement in the current taxonomy of cellular senescence, especially regarding markers that turn out to be less specific than originally expected. Likewise, a combination of both established and novel markers will increase the specificity and efficiency to detect senescence in vivo and in vitro. As already mentioned, markers for senescence-like phenotypes in post-mitotic cell entities will be a future requirement.

### Aging of Senescent Cells

In light of the age-related dynamic changes in the expression of senescence markers mentioned earlier, one might speculate that senescent cells in old tissues also undergo an aging process and acquire a dysfunctional ’aged phenotype‘, in a similar way as other cells. In the same context, accumulation of senescent cells in aging tissues might not only be causal for the aging process but also a consequence of aging, with both processes interacting through a positive feedback loop and potentiating each other (Figure 4). This possibility deserves further investigation and could also lead to new approaches to reduce the negative impact of cellular senescence in aging tissues.

## 4. Beneficial Functions of Senescence and Aging

As summarized in Figure 4, both aging and senescence are not only detrimental for the organism, but they also seem to play beneficial roles during development and in response to stress and pathologic events.

### 4.1. Embryonic Patterning, Tissue Regeneration and Repair

Chronic excessive and aberrant accumulation of senescent cells in tissues and organs affects regenerative capacities and creates a pro-inflammatory environment that influences the onset and progression of several aging-associated diseases [5,41]. However, the acute induction of cellular senescence may play beneficial roles beside tumor suppression. During development, programmed senescence is accompanied by the removal of senescent cells and tissue reshaping, a process regulated by p21^Cip1^, the TGF-β/SMAD and PI3K/FOXO pathways, but being independent of DNA damage, p53, or other cell-cycle inhibitors [129]. Accordingly, loss of senescence due to the absence of p21^Cip1^ resulted in developmental abnormalities in mice. In general, senescence is identified to be a relevant and physiological mechanism during embryonic development [130].

Next to tissue and organ development, beneficial aspects of senescence are now also disclosed for metabolic processes. Although p16^Ink4a^-induced senescence in pancreatic β-cells reinforces hallmarks of senescence, it co-stimulated insulin-secretion and mitochondrial activity, thereby improving glucose homeostasis, a process partly dependent on the activity of mTOR [131]. Such results further indicate that there is a bidirectional positive relation between insulin/IGF and senescence.

Cellular senescence also participates in tissue repair (Figure 4). In response to liver injury, activated murine hepatic stellate populations proliferate and thereby stimulate SASP components represented by extracellular matrix-degrading enzymes, recruit immune cells, and restrict fibrosis progression [132]. In contrast, senescence might also impede the regenerative liver response as exemplified in an acute acetaminofen-mediated injury model [133]. Inhibition of TGF-β secretion as part of the SASP suppressed paracrine senescence and restored the regenerative liver response. The role of senescence in liver injury and regeneration might, therefore, depend on additional factors such as cell type and lesion entity [42]. Senescent fibroblasts and endothelial cells in mice were found to promote the healing of skin lesions [134], whereas senescent cell removal compromised skin repair in mice. The seeming contradiction of impaired wound healing in old individuals might result from an anti-regenerative inflammatory and T-cell-mediated immune response [135,136]. Notably, the factors that guide immune clearance of senescent cells are still understudied. Although senescent cells are regularly eliminated by CD4^+^ T cells, neutrophils and macrophages, the key recognition patterns that initiate immune clearance cascades are yet to be defined. Likewise, overexpression of the cell surface marker DPP4 by senescent human fibroblasts reinforces antibody-directed senolysis [136]. The identification of cell and tissue type-specific surface markers and regulatory mechanisms that foster immune recognition and restrict immune subversion of senescent cells and the development of targeted antibody strategies might afford a breakthrough not only in tumor treatment but also in the prevention and therapy of neurodegenerative and other disease entities.

A further aspect positively regulated by senescence relates to cellular plasticity. A study by Ritschka and colleagues demonstrated that exposure to the SASP released from MSCV- or *Hras*^V12^-infected primary keratinocytes provides regenerative signals that induce pro-regenerative stemness in mouse keratinocytes (Figure 4). By contrast, prolonged or aberrant exposure to the SASP leads to paracrine senescence and impaired regenerative capacity [137]. Thus, exposure time to the SASP might decide over its tissue-specific deleterious or beneficial effects.

### 4.2. The Beneficial Effects of Aging

In contrast to such pleiotropic beneficial aspects assigned to the process of senescence, a physiological role of aging in the context of cellular and biological functions has not yet been formulated [2] (Figure 4). If we assume that aging already starts before birth, it can be considered simply a developmental stage, required to complete the evolutionary program associated with species-intrinsic biological functions such as reproduction, survival, and selection. More comprehensively, at the species level, aging could represent a means to prevent overcrowding of defined environments and ‘saving resources for group benefit’, or to promote evolutionary changes by accelerating the turnover of generations [138,139]. Furthermore, in the context of the aforementioned evolutionary theory of aging, increased post-reproductive survival and decline in juvenile mortality with age have been explained by an intergenerational transfer effect [140]. Thus, it relies on resources or adaptive strategies passed between individuals, whereby post-reproductive females make substantial contributions to their descendants by intergenerational parental care.

## 5. Epigenetic Mechanisms in Cellular Senescence and Aging

Beside common effectors involved in replicative senescence and aging including p53 [110] and mTOR [96] mentioned above, there is a growing perception that epigenetic modifications are also shared between both processes under in vitro and in vivo conditions [141,142] (Figure 1). Since genetics only explains 20%–30% of the aging process [143], recent studies are now focusing on alterations of the epigenetic landscape.

One striking finding is the altered cytosine methylation pattern of cytosine-guanine dinucleotides, called CpG sites, in the human and mouse genomes by aging [144,145,146,147]. These changes very accurately correlate with and thus represent a multivariate indicator of chronological age [148,149]. On this basis, the so-called epigenetic clock reflecting methylation levels of 353 CpGs, was established for humans [148], which measures cellular age in different tissue types with a significantly higher accuracy than classical aging markers such as telomere length. Variations in the heterogeneity of DNA methylation patterns among cells in a certain tissue are held as ‘the ticking rate’ behind the epigenetic clock [150]. A corresponding epigenetic clock for mice, based on only three CpG sites, is also established [151]. As epigenetic aging is related to life expectancy, both in the human and mouse, it has the potential to reflect biological rather than chronological age [152]. A positive difference between epigenetic and chronological age is defined as epigenetic acceleration [150], as it occurs in Down syndrome and progeroid Werner syndrome [153,154]. On similar principles, epigenetic patterns are used for determining gestational age at the time of birth [155].

Epigenetic aging also applies to cells in vitro, which even retain the memory of their epigenetic age in the initial culture, revealing that the epigenetic clock continues to tick outside of an in vivo tissue context [148,150].

Similarly to aging, replicative senescence exhibits specific signatures of DNA methylation [93,141,156], entailed by massive epigenetic DNA remodeling. As such repatterning has been linked to enhanced plasticity [137,157], it might underlie a reprogramming towards epigenetic features of immature cells [158].

The concept of an epigenetic clock was recently used to identify the genetic relationship between aging and senescence in isogenic cells in vitro, when senescence was induced, e.g., by multiple stressors including exhaustive replication, oncogene over-expression or radiation-induced DNA damage [72]. In contrast to RS and OIS, senescence induced by DNA damage was not accompanied by an epigenetic aging pattern, although both RS and OIS activate the DDR-pathway. However, in cases where, e.g., DNA damage-induced differentiation and senescence of neural stem cells is not accompanied by DDR, epigenetic mechanisms might be involved in senescence conversion and maintenance [159]. Thus, the epigenetic markers are sufficient to distinguish cellular aging from senescence, at least under certain in vitro conditions, and highlight the fact that aging and senescence in vitro are clearly distinct.

Apart from methylation profiles, other epigenetic markers, such as histone acetylations [160], will contribute to better define and differentiate aging and senescence. Histone H3 and H4 hypoacetylation after downregulation of p300 histone acetyltransferase also induce senescence. This mechanism is independent of the activation of p53, p21^Cip1^ and p16^Ink4a^, as shown for human diploid fibroblasts (HDF) immortalized through the expression of the human telomerase reverse transcriptase (hTERT) enzyme [161]. Under these conditions, replicative stress proceeds without DNA damage and checkpoint activation, leading to an abrupt G_2_/M cell cycle arrest, within one single cell cycle. Whether such a mechanism is relevant for cellular senescence in primary cells during physiological aging requires further investigation.

## 6. Reversibility of Aging and Senescence

Apart from yet unknown causality, the question of whether aging is reversible or not is unanswered (Figure 1). Equally uncertain is the notion of a reversible senescence process in individual tissues, which would have to rely on a cellular state conversion instead of elimination by senolysis. Such an issue might have consequences due to the role of senescence in tumor suppression [162]. Likewise, in a recent study, Leikam and colleagues illustrated that a state of OIS can be overcome in differentiated cells and give rise to transformed progeny of cells that trigger aggressive tumor formation [163].

Currently, two intercalated strategies relying on ‘deceleration’ and ‘reprogramming’ are mainly engaged in order to counteract aging and increase longevity, both of which have been translated into scientific concepts and applications. These concepts also presuppose the belief that aging is at least to a certain extent regulated by external or genetic and transcriptional factors accessible for manipulation and intervention. The strongest ‘deceleration’ or delay has been achieved by the paradigm of a highly conserved adaptive stress response elicited by calorie restriction, implicating pleiotropic metabolic, anti-oxidative and anti-inflammatory aspects [164]. Similarly, ‘reprogramming’ in terms of targeted gene modulation, e.g., via the regulation of sirtuins or interference with mTOR-related pathways, which are also effectors in calorie restriction, has a proven impact on the aging process and lifespan [164,165,166]. However, as ‘reprogramming’ decelerates rather than countermands aging, a terminology that clearly separates the process of rejuvenation from a slowing-down strategy of the aging process, as recently suggested by Galkin and colleagues [14], would be helpful in the assessment of a true reversibility of aging. According to these authors, rejuvenation should be understood as a reprogramming process that converts an old organism to an embryonic state. In terms of strategies that do not fully rejuvenate back to an embryonic phenotype, as is currently evident for rapamycin, calorie restriction, senolytics, and heterochronic parabiosis, there is still a need for adequate biomarkers that would reliably allow quantification of the degree of rejuvenation [14]. Although heterochronic parabiosis, defined as the shunting of the systemic circulation of syngeneic animals at different ages, has been proven to prolong the lifespan of the aged organism, the underlying non-genetic, apparently blood-derived factors still have to be deciphered, and a proof of principle in humans, e.g., by blood transfusion, is still not empirically confirmed. Moreover, the conclusion of ‘preventive reprogramming’ starting already at a young age to achieve efficiency might collide with the concept of aging being seen as an indelible relic of a developmental growth program, as such a strategy would possibly trigger pathology instead of delaying the manifestation of an aging phenotype. Interestingly, a recent study by Olova et al. provides evidence that in human iPSCs the kinetics of genetic aging are uncoupled from that of epigenetic aging, thus identifying a suitable time frame for putative rejuvenation, whilst avoiding cancer risk [167].

The stimulation of cell type- and tissue-specific regenerative and cellular replacement potential might add up to modulate the kinetics of aging [14]. Although sophisticated tissue and organ replacement strategies, including achievements based on the Yamanaka transcription factors and iPSCs [168,169], have been developed, which even abandon interspecies borders [170], such attempts do not significantly contribute to the molecular understanding of the aging process itself and thus were not discussed in the context of this review.

Another means reported to achieve rejuvenation is the overexpression of the enzyme telomerase in aged organisms. Telomerase activity is high during embryonic development but declines rapidly after cell and tissue differentiation. Overexpression of telomerase has been shown to reverse several aging phenotypes in multiple tissues including the CNS and to increase lifespan [171]. How far these effects are attributable to telomere length stabilization, or mediated by telomerase-independent functions [172] still has to be specified. Since most cells do not divide, as critically discussed before, accumulating damage at deeper sites of the telomeres and thus telomere insufficiency in general rather than metric telomere shortening might contribute to the manifestation of an aging phenotype over lifetime. However, whether telomere replenishment by telomerase can compensate for telomere dysfunction apart from telomere shortening is yet unclear.

Further strategies achieving genetic and epigenetic manipulation, e.g., in the scope of SNV load, methylation patterns and mobile element expression might also raise the potential to decelerate, halt or even reverse aging phenotypes, at least to a certain extent.

## 7. Conclusions and Perspectives

### 7.1. Conclusions

Cellular senescence is an established hallmark of aging, and the accumulation of senescent cells in aged tissues is considered a driver of the aging process. However, due to inconsistent application of the terminology of ‘aging’ and ‘senescence’, and the important novel finding that post-mitotic cells acquire a senescence-like phenotype in spite of their non-replicative nature, a novel interpretation of what senescence subsumes in terms of molecular determinants and biological functions appears overdue. Accordingly, based on the different aspects either established or yet nascent with regard to the origin and nature of senescence, we propose that the current definition of cellular senescence as a mechanism leading to irreversible cell-cycle arrest in replicative cell moieties should be revisited to include an analogous state described in post-mitotic cells, which here we termed ‘amitosenescence’ particularly for post-mitotic neurons. Such reconsideration is highly relevant as it could change our view on the impact of senescence on aging, and on the feasibility and efficiency of senolytic approaches in the context of neurodegenerative disorders and other age-related degenerative pathologies. However, to succeed with such concepts, beyond a descriptive terminology, progress in our perception of aging and senescence as representing identical, autonomous, or separate but interacting processes is demanded. Moreover, to achieve a better comprehension of the origin of aging, we crystalize four contrasting concepts of aging implementing knowledge from intensive research grown during the last years in this field that is: 1) the stochastic causative approach, 2) the pseudo-programmed causative concept, 3) the programmed causative view and 4) the stochastic causative, but programmed response paradigm (Figure 2). Each of these theories contributes to, but still fails to provide, a final conclusive definition for aging and thus demands further efforts. On the way to improve our stratification strategies, there is a particular need for novel sensitive and specific biomarkers, which can relate to, for example, cellular, molecular, genetic and epigenetic aspects. Likewise, the remarkable precision of the epigenetic clock to operate as a chronometer might serve as a new promising tool on the way to better define current aging paradigms, as epigenetic mechanisms seem to determine the cellular fate towards a senescent phenotype independently of chronological aging. Moreover, the crystallization of genetic signatures of aging defined on a personalized rather than universal level might bring forth the identification of subtle aging characteristics and help estimate their relation with functional deficiencies and pathologies.

Along with such evidence, we support the notion that cellular senescence and aging are part of a cellular interactome, but with the implication of independent mechanisms. The detrimental effects of senescent cells in aged tissues are, thereby, assumed to arise partly from their aberrant accumulation and a dysfunctional aging phenotype acquired by these cells as a result of the altered aging environment.

As recent studies illustrate novel physiological roles of cellular senescence, it will be further relevant to specify the consequences of cellular senescence in a cell type- and context-specific manner.

### 7.2. Perspectives

As mentioned above, some work supports the notion that senescence is also a physiological process adding to tissue homeostasis with beneficial effects during embryonic development, metabolism, regeneration and plasticity, and probably others, which will be described in upcoming studies. These novel findings pose a problem for proposed therapeutic approaches aimed at the clearance of senescent cells via so-called senolytics, drugs that provoke apoptosis in senescent cells while sparing non-senescent entities [45]. Many available senolytic drugs target only subsets of senescent cells without discriminating among beneficial and deleterious senescence programs [41]. Moreover, there is currently no clear opinion about the cellular target populations that might provide the strongest benefit by senolytic elimination [62,173]. Therefore, studies that specify the contribution of different cell entities to senolytic drug-mediated effects in individual pathological environments and rule out adverse off-target effects of senolytic treatment regimens are still required in order to achieve the expected efficiencies [128]. Such specifications will also help to answer unequivocally the question of whether some nominally post-mitotic cell populations such as neurons are subjected to cellular senescence or not.

Unexplored in this context is the contribution of a phenotypically re-induced atypical cell cycle in nominally post-mitotic neurons [174]. The concept of unscheduled cell cycle re-entry has now been consolidated for many age-related neurodegenerative disorders and is estimated to occur in up to 13%–40% of neurons under stress conditions [175]. Until recently it was assumed that such atypical cell-cycle activity will remain abortive at the latest in the S/G_2_ phase and finally end up in a delayed process of apoptosis rather than the completion of cytokinesis. However, a recent work by Walton and colleagues addresses the prospective of a full ‘neuromitotic’ event based on a complex genetic and pharmacologic intervention, involving 1) cell-cycle induction by overexpression of a CyclinE/Cdk2 fusion construct, 2) apoptosis prevention via p53 inhibition, 3) G_2_ checkpoint abrogation through the inhibition of Wee1 kinase, and 4) topoisomerase-2α overexpression to aid sister chromatid decatenation and cytokinesis [176]. The authors conclude from their in vitro approach that differentiated neurons are principally equipped with functional cell-cycle machinery, the progression of which seemingly follows a genuine sequence comparable to that intrinsic of replicative cells. Accordingly, in our own studies, under ALS-like conditions using mice carrying a *hSOD1^G93A^* mutation, we found DNA content alterations indicative of an S/G_2_ phase recapitulation, coincident with altered transcript and protein levels of several positive and negative cell-cycle regulators (unpublished). Interestingly, similar DNA content imbalance was also observed in cortical neurons under healthy aging conditions (unpublished). If neurons are indeed competent to fully replicate their DNA, the question arises of whether such an event will be singular or imply the capacity for repeated cell cycles, and whether the cycle number completed without being driven into apoptosis would be numerous enough to impact telomere length and to induce a senescent phenotype. A further question is whether senescence markers found in post-mitotic neurons might result from the abortion of an unconventional rather than truly mitotic cell cycle. Indeed, multi-nucleation, a parameter recently utilized to identify neurons passing through an aberrant cell cycle without cell division [176], is a common signature of cellular senescence caused by oncogenic or replication stress in vitro. Thus, additional studies are required to further specify the presence, stability and functional relevance of intermediate cell-cycle states putatively intercalated between ultimate G_0_ arrest and mitosis in mature neurons, also under in vivo conditions. Functional re-interpretation of atypical cell cycle events might address the question of whether cell cycle re-entry will be re-assigned to compensate for the limitation of neurons in their DNA repair repertoire, as they are normally incompetent to perform homologous recombination in order to restitute double strand breaks. Such a possibility is fueled by the recent discovery of an RNA-templated, HR-related DNA recombination repair pathway independent of replication, as identified for the G_0_/G_1_ phase of differentiated cells in a Cockayne model of transcription-coupled nucleotide excision repair (TC-NER) deficiency, and further evidenced for neurons [177,178]. Moreover, as cellular senescence is triggered by DNA damage, re-addressing aberrant cell-cycle re-entry in the context of DDR-induced senescence might provide an alternative mechanism on how neurons profit from or undergo senescence. This raises the question of whether removal of neurons with atypical cell-cycle activity will influence the aging process.

An alternative approach is to reduce the negative effects of the SASP, e.g., by inhibiting the pleiotropic transcription factor NF-κB, a major driver of the SASP. Interestingly, some life-extending compounds, such as rapamycin, resveratrol, and metformin, have been shown to decline SASP factors with variable efficiencies [96,179]. However, this approach has also experienced several drawbacks since most of the compounds entail severe toxicities when used for a prolonged time. Apart from expediting the SASP, NF-κB has now been discovered to propagate several other major aspects of aging and, thus, its inhibition appears to be a promising target to counteract inflamm-aging, immuno-aging and to positively influence aging phenotypes and lifespan [112]. Its interference even confers the potential to add up classical antiapoptotic [180] and pro-regenerative effects [181]. However, considering the strong dependency of NF-κB effects on the cellular and pathophysiological context and the dimers activated, such intervention would require a further refined stratification according to the subunits and cell types addressed.

The findings delineated with respect to the role of epigenetic changes in cellular senescence and aging also open up a new avenue for therapeutic interventions, as suggested by the possibility of epigenetic reprogramming of senescent cells [112]. This approach is particularly tempting, as it considers the possibility to ’rejuvenate‘ cells undergoing replicative senescence and to set back chronological aging [112]. Overcoming the risk of cancer development will be the primary challenge in achieving rejuvenation.

## Abbreviations

AASP	Aging-associated secretory phenotype
ALS	Amyotrophic lateral sclerosis
CCL-2	C-C motif chemokine ligand 2
Cdk/CdkI	Cyclin-dependent kinase/Cyclin-dependent kinase inhibitor
cGAS	Cyclic guanosine monophosphate – adenosine monophosphate synthase
CSF	Colony-stimulating factor
CXCL	Chemokine C-X-C motif ligand
DDR	DNA damage response
DNA-SCARS	DNA segments with chromatin alterations reinforcing senescence
DPP4	Dipeptidyl peptidase-4
FoxO	Forkhead box protein O
GH	Growth hormone
HLA	Human leukocyte antigen
HR	Homologous recombination
IGF-1	Insulin-like growth factor-1
iPSCs	Inducible pluripotent stem cells
LINE-1	Long interspersed nuclear element-1
LTR	Long terminal repeats
MAPK	Mitogen-activated protein kinase
MMP	Matrix-metalloprotease
mTOR	Mechanistic target of rapamycin
NF-κB	Nuclear factor κB
NK cell	Natural killer cell
OIS	Oncogene-induced senescence
PI3K	Phosphoinositid-3-kinase
PML	Promyelocytic leukemia protein
ROS	Reactive oxygen species
RS	Replicative senescence
SA-β-Gal	Senescence-associated-β-galactosidase
SAHF	Senescence-associated heterochromatic foci
SASP	Senescence associated secretory phenotype
SIPS	Stress-induced premature senescence
STH	Somatotropin
STING	Stimulator of interferon genes
TC-NER	Transcription-coupled nucleotide excision repair
